# COVID-Inconfidentes: how did COVID-19 and work from home influence the prevalence of leisure-time physical inactivity? An analysis of before and during the pandemic

**DOI:** 10.1186/s12889-022-14145-1

**Published:** 2022-09-16

**Authors:** Samara Silva Moura, Luiz Antônio Alves Menezes-Júnior, Ana Maria Sampaio Rocha, Luciano Garcia Lourenção, Júlia Cristina Cardoso Carraro, George Luiz Lins Machado-Coelho, Adriana Lúcia Meireles

**Affiliations:** 1 Nutrition School, Post-Graduate Program in Health and Nutrition, Federal University of Ouro, Preto, Diogo de Vasconcelos 122, Ouro Preto, Minas Gerais Brazil; 2 Epidemiology Laboratory, Medical School, Federal University of Ouro Preto, Ouro Preto, Minas Gerais Brazil

**Keywords:** Physical inactivity, COVID-19, Social isolation, Health research

## Abstract

**Background:**

The COVID-19 pandemic has brought drastic changes to the lives of the global population. The restrictions imposed by government agencies impacted the daily lives of citizens, influencing several health behaviors, such as physical activity (PA). Thus, the present study aimed to assess the prevalence of physical inactivity (PI) and its associated factors before and during the COVID-19 pandemic.

**Methods:**

A population-based household seroepidemiological survey was conducted in two Brazilian municipalities located in the state of Minas Gerais, in which 1750 volunteers were interviewed between October and December 2020. Face-to-face interviews were conducted using a structured questionnaire in an electronic format. The moments considered for the PI analysis were M0 (before the pandemic), M1 (from March to July 2020), and M2 (from October to December 2020). Descriptive statistics and univariate and multivariate logistic regression were used to examine the factors associated with PI before (M0) and during the pandemic (M1 and M2).

**Results:**

The prevalence of PI was higher in the first months of the pandemic (M1) (67.3%; 95% confidence interval (CI): 62.4–71.9) than in the months from October to December 2020 (M2) (58.7%; 95% CI: 52.8–64.3); however, at both times (M1 and M2), PI was more prevalent than in the period before the pandemic started (M0) (39.7%; 95% CI: 35.6–43.8). Individuals who were overweight, obese, and had low educational levels were more likely to be physically inactive. At both M1 and M2, individuals who worked at a work from home were less likely to have PI.

**Conclusions:**

The results suggest that the COVID-19 pandemic negatively influenced PA, substantially increasing the prevalence of PI. The determinants associated with PI were education, body mass index, and work from home.

## Background

Severe acute respiratory syndrome coronavirus 2 (SARS-CoV-2), the causative agent of COVID-19, is part of a group of viruses responsible for causing acute respiratory syndrome. Infection caused by this virus has a clinical spectrum, ranging from asymptomatic to severe, and is associated with significant morbidity and mortality rates [1]. Due to the speed of spread of the virus and the high rate of infection, concomitant with the lack of knowledge of specific therapies, strategies have been established to mitigate the spread of the virus and reduce its impact [2]. Thus, one of the main prevention measures recommended by the World Health Organization (WHO) and adopted by governments is social restriction [3, 4], aimed at minimizing the exponential growth of infected people, avoiding deaths, and not generating a collapse of health systems [2].

Despite the benefits related to the decrease in viral circulation, social restriction promoted a sudden and drastic impact, both in the economy, with a reduction in household income, and in aspects related to health, such as eating habits, sleep quality, sedentary behavior, and physical activity (PA) practice [5]. Specifically, regarding PA practice, in the first months of the COVID-19 pandemic, the closure of several commercial establishments, such as gyms, and of public spaces open for PA practice, such as squares and parks, may have led to an increase in the prevalence of physical inactivity (PI) in the population. According to the current recommendations (≥ 150 min of moderate PA or ≥ 75 min of vigorous activity), PI refers to the performance of insufficient amounts of moderate to vigorous intensity activities [6, 7] and may generate a negative impact on physical and mental health [8] and favor an increase in the prevalence of chronic diseases, such as coronary heart disease, colon and breast cancer, hypertension, stroke, type 2 diabetes, and osteoporosis [9, 10].

The effects of COVID-19 on PA have begun to be studied [11–19]. Prolonged staying at home demonstrates reduced urban mobility worldwide, in addition to the increased prevalence of PI [11]. In Brazil, a cross-sectional study that evaluated 43,995 adults during the pandemic period indicated that physically active subjects became inactive during social restriction. In Spain, a study evaluating 3,800 healthy adults found that especially young people, students, and very active men significantly decreased self-reported daily PA and considerably increased the time of sedentary behavior during confinement [12]. Corroborating these results, a study conducted with hypertensive elderly using accelerometers to check for changes in PA before (January to March 2020) and during (June 2020) the COVID-19 pandemic. A significant reduction in moderate PA was observed, as well as an increase in the time of sedentary behavior during this period. The authors concluded that social restriction caused unhealthy changes in behaviors, and the patterns adopted in this population may have several cardiovascular and metabolic implications, being a group at risk and already prone to chronic disease [13]. There are numerous studies related to the negative impacts of social restriction in the COVID-19 pandemic [14–18].

Thus, a better understanding of how the COVID-19 pandemic influenced PA/PI change over time, which is less understood. Making it of great relevance to the civil, scientific and political community, as well as health officials, because it provides data for health planning. With this, it becomes possible to develop strategies, actions, subsidies, and programs to formulate policies aimed at physical and emotional well-being, promoting quality of life, and enabling the mitigation and reduction of economic expenditures in the health system. Therefore, this study aimed to assess the prevalence of PI and its associated factors before and during the COVID-19 pandemic.

## Methods

### Design and sample

The COVID-Inconfidentes study is a population-based seroepidemiological household survey conducted between the months of October and December 2020 in two Brazilian municipalities (Ouro Preto and Mariana), located in the state of Minas Gerais, in the Iron Quadrangle region, which is an area with one of the largest iron ore reserves in the world and is economically important. Face-to-face interviews were conducted, in the homes of the selected individuals, using a structured questionnaire in an electronic format [19].

The sample size calculation considered the 2010 population census for the urban area of the municipal headquarters of each municipality, 95% confidence level, estimated infection by SARS-CoV-2 from 3 to 10%, design effect equal to 1.5, and 20% of recomposition, considering losses due to refusals, absence of the resident drawn, and the existence of closed households during the visit. The sample was calculated using the OpenEpi program (https://www.openepi.com/Menu/OE_Menu.htm), totaling 732 interviews for each municipality. We used conglomerate sampling in three stages: census sector (selected with probability proportional to the number of households), household (selected from a systematic sampling), and resident (randomly selected through the application Sorteador de Nomes®) [19].

The sample weight of each selected unit (census sector, household, and individual) was calculated to correlate with the 2019 population projections (DATASUS) [20]. Adjustments were applied in this calculation to compensate for interview losses due to non-response. Further details on the sample calculation and field logistics are described by Meireles et al. [19].

The inclusion criteria for the study were adults (aged 18 years and older) with permanent residence in the urban areas of Ouro Preto and Mariana, cognitive ability, and venous access for serological testing. The exclusion criteria were individuals under 18 years old, residents of social centers and long-stay institutions, quarantine due to current diagnosis of severe acute respiratory syndrome coronavirus-2 (SARS-CoV-2) infection, cognitive impairment, and infeasibility of collecting blood samples due to difficult venous access.

### Outcome variable: leisure-time physical inactivity

Participants were asked about PA during leisure time at different times related to the pandemic. For the moment before the pandemic (Moment 0 [M0]), we asked, “Before the pandemic (March/2020) did you practice physical exercise? (1) No; (2) Yes.” Then, they were asked about the moment referring to the first months of the pandemic, referred to as Moment 1 (M1), “During the first months of the pandemic (March to July/2020), did you participate in physical exercise? (1) No; (2) Yes.” Finally, they were asked about the moment of data collection, referred to as Moment 2 (M2), “Do you currently (October-December 2020) participate in any type of physical exercise? (1) No; (2) Yes.”

Individuals who self-reported participating in PA during leisure time were classified as physically active, and those who said they did not participate in PA during leisure time were classified as physically inactive.

### Explanatory variables

We considered socioeconomic, nutritional status, and COVID-19-related variables as explanatory variables. The socioeconomic information analyzed were as follows: gender, age group (18–34 years; 35–59 years; ≥ 60 years), race (white; non-white), marital status (widowed, divorced, and single were categorized as single; married/stable union categorized as married), current income (≤ 2 minimum wages; > 2 to ≤ 4 minimum wages; > 4 minimum wages), and level of education (< 9 years of study; ≥ 9 years of study).

The nutritional status was assessed by body mass index (BMI), calculated from self-reported weight and height. The BMI was classified as underweight (BMI < 18.5 kg/m2 if < 60 years or BMI < 22.0 kg/m2 if ≥ 60 years), eutrophic (BMI 18.5–24.9 kg/m2 if < 60 years or BMI 22, 0–27.0 kg/m2 if ≥ 60 years), overweight (BMI ≥ 25.0 kg/m2 if < 60 years or BMI ≥ 27.0 kg/m2 if ≥ 60 years), and obese (BMI ≥ 30 kg/m2 if < 60 years) [21, 22].

Regarding COVID-19, the following variables were included: work format during the pandemic, social withdrawal, COVID-19 symptomatology, and anti-SARS-CoV-2 serological examination.

“In addition, we also assessed the work routine during social restriction. Therefore, individuals who were not working at the time of data collection were classified as “no work”. And among those who did work, those in whom all work activities were being performed in the work environment were classified as “no work from home”, and those who were working partially or completely from home as “work from home”.

Questions related to the COVID-19 pandemic were also evaluated, such as presenting at least one symptom in the last 15 days (fever, feeling feverish, palpitation, diarrhea, sore throat, cough, difficulty breathing, vomiting, skin rashes, unusual tiredness, ageusia, and anosmia). Responses were categorized into the presence (one or more symptoms) and absence of symptoms.

Social restriction was assessed using the question, “Are you currently on social restriction? (1) No; (2) Yes.”

Finally, for seroepidemiological evaluation of anti-SARS-CoV-2 antibodies, serum samples were obtained by venipuncture using a 7.5 mL S-Monovette® (Sarstedt) serum gel tube. The samples diagnosed by qualitative immunochromatographic method using the One Step COVID 2019® test (Guangzhou Wondfo Biotech, China), according to the manufacturer’s protocol. The seropositive results for this method are defined by the presence of two bands indicating, respectively, its performance and the non-differentiated presence of IgM/IgG anti-SARS-COV-2 antibodies [19].

### Ethical declarations

This study was approved by the Research Ethics Committee of the Universidade Federal de Ouro Preto, under protocol number 32815620.0.1001.5149. All procedures adopted in this study followed the Declaration of Helsinki and the Brazilian guidelines and norms for research involving humans.

### Data analysis

The statistical analyses considered the complex sample design using the svy command of Stata® software, version 15.0. Descriptive analysis was performed, with calculation of frequency and 95% confidence interval (95%CI) for all variables of interest. McNemar’s paired test was used to verify the change in the prevalence of PI during the three evaluations. Pearson’s χ 2 test was used to assess the possible relationship between work from home and sociodemographic factors.

Univariate logistic regression was performed to assess the factors associated with PI before the pandemic and at the two time points of the COVID-19 pandemic. Based on the results of this analysis, variables with *p* ≤ 0.20 were included in the multivariate logistic regression. A stepwise backward approach was used to choose the final model, and variables with *p* ≤ 0.05, were retained. The odds ratios (ORs) and 95% CIs were calculated.

It is noteworthy that the variables income and education showed high collinearity in this study, so we chose education, instead of income, in the multivariate model.

## Results

During the data collection period, 5,252 households were approached, of which 2,523 (48.0%) in the municipality of Mariana and 2,713 (52.0%) in the municipality of Ouro Preto. Of the total, 1,912 (36.4%) households were closed; in 1,079 (20.5%) there was refusal by the residents; in 267 (5.1%) the drawn resident was absent; and 1,762 (33.5%) residents agreed to participate in the survey, of which 764 (43.4%) in Mariana and 998 (56.6%) in Ouro Preto. A total of 1750 randomly selected participants agreed to participate in the survey.

However, 12 participants were excluded from the analyses of the present study for not completing the answers related to physical activity. The survey included 1750 individuals, with the majority being female (52.4%; 95% CI: 40.5–54.8), aged 35–59 years (45.8%; 95% CI: 41.2–50.5), non-white skin color (73.9%; 95% CI: 68.4–78.8), with less than nine years of schooling (69.1%; 95% CI: 64.3–73.6), income below two minimum wages (41.2%; 95% CI: 35.6–47.1), and eutrophic (41.1%; 95% CI: 34.9–47.7). Most self-reported being in social restriction (86.2%; 95% CI: 82.0–89.5), had no symptoms of COVID-19 (70.6%; 95% CI: 65.4–75.3), and were seronegative for the COVID-19 test (94.3%; 95% CI: 92.3–95.8), according to Table 1.

**Table 1:** General sociodemographic characteristics of the study sample. COVID-Inconfidentes, 2020

Variables	% (95% CI)
**Sex**	
Male	47.6 (40.5–54.8)
Female	52.4 (45.2–59.4)
**Age group**	
18–34 years	35.1 (30.8–39.7)
35–59 years	45.8 (41.2–50.5)
≥ 60 years	19.1 (15.7–23.0)
**Declared Skin color**	
White	26.1 (21.2–31.6)
Not white	73.9 (68.4–78.8)
**Education**	
≥ 9 years	69.1 (64.3–73.6)
< 9 years	30.9 (26.4–35.7)
**Family Income**	
≤ 2 MW	41.2 (35.6–47.1)
> 2 a ≤ 4 MW	31.4 (26.3–36.9)
> 4 MW	27.4 (22.3–33.1)
**Nutritional status**	
Underweight	2.3 (1.5–3.5)
Eutrofic	41.1 (34.9–47.7)
Overweight	37.0 (29.5–45.1)
Obesity	19.6 (16.2–23.4)
**Social Restriction**	
No	13.8 (10.5–17.9)
Yes	86.2 (82.0–89.5)
**Symptomatology COVID-19**	
Absence of symptoms	70.6 (65.4–75.3)
Presence of symptoms	29.4 (24.6–34.5)
**COVID-19 Test**	
Soronegative	94.3 (92.3–95.8)
Soropositive	5.7 (4.2–7.6)
**Work routine during social restriction**	
No work from home	32.0 (27.5–36.9)
No work	47.843.4–52.3)
Work from home	20.2 (16.-26.8)

Minimum wage; *CI* Confidence intervals (95%). “Family income: minimum wage value (2020): BRL 1045.00 ≈ USD 194.25 (1 USD = 5.3797 BRL)

**Fig. 1:**
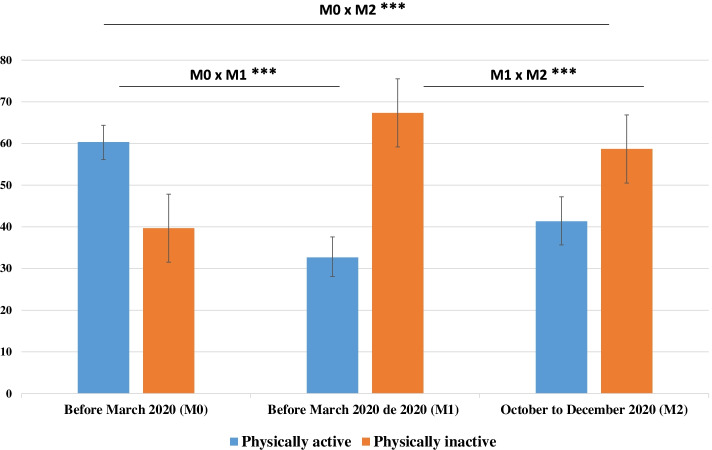
Prevalence of physical inactivity at moments M0 (before the pandemic), M1 (March to July 2020) and M2 (October to December 2020). (COVID-Inconfidentes, 2020). *p < 0.05, **p < 0.01, ***p < 0.001. McNemar’s paired test

Figure 1 shows the prevalence of PI according to the time points investigated. We observed that 39.7% (95% CI: 35.6–43.8) of the individuals were inactive before the pandemic (M0). There was a 69.5% increase in the prevalence of PI at M1 (67.3%; 95% CI: 62.4–71.9) and a 47.8% increase in the prevalence of PI M2 (58.7%; 95% CI: 52.8–64.3) compared to that during the period before the pandemic (M0) (*p* < 0.001). When we evaluated the moments M1 and M2, we observed that the prevalence of PI was 21.7% higher in M1 than in M2 (*p* < 0.001).

In Table 2, we observe the prevalence of PI according to the moments investigated and the crude OR of the explanatory variables and PI. No associations were observed between PI and the variables gender and related to COVID-19 (social withdrawal, symptomatology, and testing positive for COVID-19). On the other hand, age ≥ 60 years (M0: OR = 1.83; 95%CI:1.05–3.20; M2: OR = 3.09; 95%CI:1.47–6.52) and age 35–59 years (M1: OR = 1.58; 95% CI: 1.01–2.47; M2: OR = 1.67; 95% CI: 1.08–2.58), education level (M0: OR = 2.53; 95% CI: 1.49–4.28; M1: OR = 2.38; 95%CI:1.45–3.93; M2: OR = 2.88; 95% CI:1.88–4.43), income > 4 MW (M0: OR = 0.26; 95%CI: 0.13–0.50; M1: OR = 0.56; 95% CI:0.33–0.95; M2: OR = 0.47; 95% CI: 0.28–0.78) and income > 2 a ≤ 4 MW (M0: OR = 0.58; 95% CI: 0.34–0.96). For BMI Overweight (M0: OR = 1.79; 95% CI: 1.10–2.90; M1: OR = 2.28; 95% CI: 1.22–4.23; M2: OR = 2.08; 95% CI: 1.19–3.61) and BMI Obesity (M1: OR = 2.57; 95% CI: 1.53–4.32; M2: OR = 2.15; 95% CI: 1.25–3.74) were associated with PI at different time points. It is noteworthy that self-reported skin color (M2: OR:1.78; 95% CI:1.13–2.80) and marital status (M2: OR: 0.55; 95% CI: 0.33–0.90) variables were associated only with M2. Furthermore, working at a work from home was associated with PI at M1 (OR: 0.45; 95% CI: 0.24–0.85) and M2 (OR: 0.51; 95% CI: 0.29–0.88).

**Table 2:** Univariate analysis of factors associated with physical inactivity: before and during two moments of the pandemic

Variables	PI (M0) (Before March 2020)	P value	PI (M1) (March to August 2020)	P value	PI (M2) (October to December 2020)	P value
	**OR (95% CI)**		**OR (95% CI)**		**OR (95% CI)**	
SOCIODEMOGRAPHIC						
**Sex**						
Male	1.00		1.00		1.00	
Female	1.27 (0.87–1.85)	0.210	1.45 (0.93–2.26)	0.093	1.25 (0.77–2.04)	0.349
**Age group**						
18–34 years	1.00		1.00		1.00	
35–59 years	1.53 (0.79–2.98)	0.198	**1.58 (1.01–2.47)**	**0.044**	**1.67 (1.08–2.58)**	**0.021**
≥ 60 years	**1.83 (1.05–3.20)**	**0.033**	1.77 (0.96–3.26)	0.066	**3.09 (1.47–6.52)**	**0.003**
**Skin color declared**						
White	1.00		1.00		1.00	
Not white	1.45 (0.95–2.25)	0.084	1.34 (0.78–2.30)	0.283	**1.78 (1.13–2.80)**	**0.012**
**Marital Status**						
Married	1.00		1.00		1.00	
Not-married	0.92 (0.63–1.35)	0.690	0.74 (0.40–1.35)	0.335	**0.55 (0.33–0.90)**	**0.019**
**Education**						
> 9 years	1.00		1.00		1.00	
< 9 years	**2.53 (1.49–4.28)**	**0.001**	**2.38 (1.45–3.93)**	**0.001**	**2.88 (1.88–4.43)**	** < 0.0001**
**Family Income**						
≤ 2 MW	1.00		1.00		1.00	
> 2 a ≤ 4 MW	**0.58 (0.34–0.96)**	**0.036**	0.60 (0.35–1.05)	0.078	0.76 (0.42–1.39)	0.377
> 4 MW	**0.26 (0.13–0.50)**	** < 0.001**	**0.56 (0.33–0.95)**	**0.034**	**0.47 (0.28–0.78)**	**0.004**
**Nutritional status**						
Eutrofic	1.00		1.00		1.00	
Underweight	1.26 (0.48–3.30)	0.639	1.06 (0.39–2.91)	0.905	0.86 (0.31–2.36)	0.762
Overweight	**1.79 (1.10–2.90)**	**0.019**	**2.28 (1.22–4.23)**	** < 0.001**	**2.08 (1.19–3.61)**	**0.010**
Obesity	1.51 (0.85–2.67)	0.154	**2.57 (1.53–4.32)**	**0.011**	**2.15 (1.25–3.74)**	**0.007**
VARIABLES COVID-19						
**Work routine during social restriction**						
No work from home			1.00		1.00	
No work	-	-	1.71 (0.73–1.86)	0.500	**1.64 (1.07–2.51)**	**0.021**
Work from home			**0.45 (0.24–0.85)**	**0.014**	**0.51 (0.29–0.88)**	**0.016**
**Social Restriction**						
No	-	-	1.00		1.00	
Yes			0.98 (0.55–1.75)	0.954	1.30 (0.84–2.00)	0.223
**Symptomatology COVID-19**						
Absence of symptoms	-	-	1.00		1.00	
Presence of symptoms			0.82 (0.51–1.34)	0.445	0.89 (0.55–1.46)	0.664
**COVID-19 Test**						
Soronegative	-	-	1.00		1.00	
Soropositive			0.73 (0.35–1.52)	0.404	1.18 (0.60–2.32)	0.611

*PI* Physical inactivity, *OR* Odds ratios, *CI* Confidence intervals (95%), *MW* Minimum wage. “Family income: minimum wage value (2020): BRL 1045.00 ≈ USD 194.25 (1 USD = 5.3797 BRL)

The final models for each time point are listed in Table 3. Individuals > 9 years of education when compared to those with ≥ 9 years were twice as likely to be physically inactive at the three time points evaluated (M0: OR = 2.55; 95% CI: 1.41–4.60; M1: OR = 1.83; 95% CI: 1.11–3.03; M2: OR = 1.78; 95% CI: 1.04–3.04). Regarding nutritional status, considering eutrophic individuals as reference, we verified that those with overweight were 79% to 150% more likely to be physically inactive at the three time points evaluated (M0: OR = 1.79; 95% CI: 1.38–2.81; M1: OR = 2.50; 95% CI: 1.40–4.46; M2: OR = 2.19; 95% CI: 1.30–3.67). Those with obesity were 85 to 124% more likely to be physically inactive (M1: OR = 2.24; 95% CI: 1.34–3.75; M2: OR = 1.85; 95% CI: 1.15–3.00), considering eutrophic individuals as reference. Regarding work status during the pandemic, individuals in work from home were 92 to 104% less likely to be physically inactive (M1: OR = 0.52; 95% CI: 0.30–0.89; M2: OR = 0.49; 95% CI: 0.28–0.84), considering the individuals in normal work routine as reference.

**Table 3:** Association of physical inactivity before and during two moments of the COVID-19 pandemic

	PI (M0) (Before March 2020)		PI (M1) (March to August 2020)		PI (M2) (October to December 2020)	
	**OR (95% CI)**	p	**OR (95% CI)**	p	**OR (95% CI)**	p
**Education**						
< 9 years	1.00 (Ref)		1.00 (Ref)		1.00 (Ref)	
> 9 years	**2.55 (1.41–4.60)**	**0.002**	**1.83 (1.11–3.03)**	**0.018**	**1.78 (1.04–3.04)**	**0.035**
**Nutritional status**						
Eutrofic	1.00 (Ref)		1.00(Ref)		1(Ref)	
Underweight	0.96 (0.35–2.66)	0.943	0.64 (0.23–1.78)	0.382	0.62 (0.22–1.76)	0.364
Overweight	**1.79 (1.38–2.81)**	**0.012**	**2.50 (1.40–4.46)**	**0.002**	**2.19 (1.30–3.67)**	**0.003**
Obesity	1.19 (0.70–2.05)	0.516	**2.24 (1.34–3.75)**	**0.003**	**1.85 (1.15–3.00)**	**0.012**
**Work routine during social restriction**						
No work from home			1.00 (Ref)		1.00 (Ref)	
No work			1.29 (0.77–2.20)	0.325	0.97 (0.62–1.51)	0.880
Work from home			**0.52 (0.30–0.89)**	**0.019**	**0.49 (0.28–0.84)**	**0.010**

*PI* Physical inactivity, *OR* Odds ratios, *CI* Confidence intervals (95%)

Multivariate model fitted to the best fit model by the stepwise backward technique, using those who reported practicing physical activity (PA) as the reference. All three models were adjusted for sex and age

As work from home was a relevant and understudied finding during social restriction, mainly associated with physical activity. Therefore, it investigated in more detail the profile of these individuals. We chose not to detail previously evaluated variables, even with statistical differences, since these data are already observed in other studies (Education and BMI).

In Table 4, the participants who belonged to the group that worked at a work from home were mostly female (54.3%; 95% CI: 44.4–63.8; *p* = 0.001), aged between 35 and 59 years (51.8%; 95%CI: 42.9–60.5; *p* < 0.001), non-white skin color (64.1%; 95% CI: 55.9–71.4; *p* = 0.02), underwent more than 9 years of schooling (95.4%; 95% CI: 90.6–97.7; *p* < 0.001), had income above four minimum wages (51.6%; 95% CI: 41.3–61.7; *p* < 0.001), and self-reported as being in social restriction (88.0%; 95% CI: 82.6–91.8; *p* < 0.001), according to Table 4.

**Table 4:** General sociodemographic characteristics and COVID-19 according to work routine during social restriction. COVID-Inconfidentes, 2020

**Variables**	**No work** 47.8% (95% CI:43.3–52.3)	**No work from home** 32.0% (95% CI:27.5–36.9)	**Work from home** 20.2% (95% CI:16.2–24.7)	*p value*
SOCIODEMOGRAPHIC				
** Sex**				
Male	38.5 (29.8–48.1)	62.3 (51.6–71.9)	45.7 (36.1–55.5)	
Female	61.4 (51.8–70.2)	37.7 (28.0–48.3)	54.3 (44.4–63.8)	** < 0.001**
** Age group**				
18–34 years	24.2 (18.9–30.3)	46.3 (35.4–57.5)	43.4 (34.1–53.1)	
35–59 years	40.3 (32.4–48.7)	50.2 (39.7–60.6)	51.8 (42.9–60.5)	** < 0.001**
≥ 60 years	35.5 (29.1–42.4)	3.5 (1.8–8.0)	4.8 (2.8–8.0)	
** Skin color declared**				
White	21.2 (16.8–26.5)	27.0 (18.0–38.4)	35.9 (28.5–44.0)	
Not white	78.8 (73.5–83.2)	73.0 (61.6–82.0)	64.1 (55.9–71.4)	**0.025**
** Marital Status**				
Married	56.4 (48.8–63.6)	51.1 (40.2–61.8)	47.5 (39.8–55.2)	
Not-married	43.6 (36.3–51.1)	48.9 (38.1–59.7)	52.5 (44.8–60.1)	0.293
** Living status**				
Alone	5.0 (3.2–7.6)	4.7 (2.3–9.2)	4.4 (3.0–6.2)	
Not-alone	95.0 (92.3–96.7)	95.3 (90.7–97.7)	95.6 (93.4–96.5)	0.894
** Education**				
> 9 anos	51.4 (43.4–59.2)	79.2 (71.8–84.9)	95.4 (90.6–97.7)	
Up to 9 years	48.6 (40.7–56.5)	20.8 (15.0–28.1)	4.6 (2.2–9.3)	** < 0.001**
** Family Income**				
≤ 2 MW	51.8 (43.3–60.1)	41.1 (32.3–50.5)	17.7 (11.0–27.3)	
> 2 a ≤ 4 MW	28.8 (22.9–35.5)	35.4 (26.6–45.2)	30.7 (23.5–38.8)	** < 0.001**
> 4 MW	19.4 (14.6–25.2)	23.5 (13.4–37.9)	51.6 (41.3–61.7)	
VARIABLES COVID-19				
** Symptomatology COVID-19**				
Absence of symptoms	68.5 (62.1–74.2)	76.9 (68.0–83.9)	65.8 (57.1–73.5)	
Presence of symptoms	31.5 (25.8–37.8)	23.1 (16.0–32.0)	34.2 (26.5–42.8)	0.080
** Social Restriction**				
No	9.7 (6.7–13.7)	21.0 (14.3–29.7)	12.0 (8.1–17.3)	
Yes	90.3 (86.2–93.2)	79.0 (70.2–85.6)	88.0 (82.6–91.8)	** < 0.001**
** COVID-19 Test**				
Soronegative	92.7 (89.1–95.1)	95.1 (92.2–97.0)	96.8 (94.2–98.3)	
Soropositive	7.3 (4.8–10.9)	4.9 (3.0–7.8)	3.2 (1.7–5.7)	0.058

Minimum wage; *CI* Confidence intervals (95%). “Family income: minimum wage value (2020): BRL 1045.00 ≈ USD 194.25 (1 USD = 5.3797 BRL)

## Discussion

This study presents important evidence on PI during the COVID-19 pandemic, suggesting that social restriction, although necessary, may have contributed to a higher prevalence of PI at the two time points assessed after the pandemic onset. The results revealed that before and during the pandemic, leisure-time PI was associated with lower educational attainment, overweight, and obesity. Moreover, working in a work from home was a protective factor for PI in both moments evaluated during the pandemic.

PA practice benefits different aspects of life, whether physical, mental, and social [23]. However, the pandemic caused abrupt changes, which contributed to the increase in the prevalence of PI [24]. Several studies have already shown evidence of a PI pandemic, even before COVID-19 [25], leading the WHO to launch a global action plan to encourage PA practice in June 2018, seeking a 15% reduction in PI rates by 2030 [26]. However, with the advent of the COVID-19 pandemic, there has been a worsening of this scenario due to social restriction and reduced urban mobility [24].

Our findings are corroborated by recent literature, which highlights the considerable increase in PI during the COVID-19 pandemic. The results further indicate that the rates of PI found in the study population during the pandemic are more than double the global prevalence (27.5%), as well as is higher than the rate found in other countries during social restriction [27, 28]. In China, a population-based study conducted in the early pandemic period (January and February 2020) with 12,107 participants found that 60% of the population did not meet the 150 min per week of PA in the four domains (leisure, commuting, home, and work) and were, therefore, considered physically inactive [27]. A similar condition was reported in Spain, in which self-reported PA in the four domains decreased significantly across the population during the lockdown, resulting in a reduction of 16.8% (*p* < 0.001) and 58.2% (*p* < 0.001) in vigorous PA and walking time, respectively [12]. Moreover, corroborating our results, an online survey conducted in Brazil between April and May 2020 with 43,995 adults found a prevalence of leisure-time PI of 66.6%, with 21.3% of individuals becoming inactive after the onset of the pandemic [29]. Furthermore, similar to our findings, a study conducted in adults over 50 years in the first six months of the pandemic, the authors observed a 42.7% decrease in PA for the elderly [30]. n a systematic review [31] that evaluated the effect of pandemic COVID-19 on PA, decreased PA and/or increased sedentary time was found in the population.

It is important to highlight that PA is determined by several individual, social, environmental, and political factors [32], and, in general, it may vary according to age, gender, income, and education level [33]. In the present study, we found an association between leisure-time PI and lower educational level, overweight status, and obesity, which can be considered to hinder the participation of leisure-time PA [34, 35].

It is well documented that lower education is associated with PI in general. Our findings reinforce data from the literature in which low education was reported as a factor that increases the likelihood of PI [36]. In a study by Kari et al. [33], the authors stated that a higher level of education is related to making healthier lifestyle decisions, including PA. Similar findings were found in the study by Park and Kang [37], which showed that an increase in the years of schooling in adulthood induces individuals to participate in PA more regularly. Furthermore, a direct relationship is perceived between schooling and PA that may be permeated by income, which, in turn, offers more opportunities to invest in PA [33, 38]. Furthermore, a peer-reviewed systematic review identified education as a positive determinant of PA [39].

The higher probability of occurrence of PI in overweight or obese individuals found in our study is a relevant factor because, in addition to complicating the overweight condition, it may contribute to the emergence and worsening of chronic diseases [40]. Studies prior to the COVID-19 pandemic have stated that a higher level of PA is more prevalent in individuals with lower BMI, and the reverse is also true, that is, subjects with higher BMI are less likely to meet the guidelines for moderate to vigorous PA [41–44]. Although the results of our study cannot demonstrate causal relationships, it is well-established in the literature that participating in PA prevents obesity and metabolic diseases arising from overweight status [40, 45, 46]. These findings are alarming and require the implementation of actions and public policies to encourage PA engagement, as the restrictions imposed to mitigate the circulation of SARS-CoV-2 contributed to weight gain in the population [47–49] and, in the current scenario, the control of overweight status or obesity becomes more challenging. Moreover, the high prevalence of overweight status and obesity, along with reduction in PA practice during the pandemic, increases vulnerability to several diseases, including COVID-19, leading to increased costs and a possible crisis in the health care system [50, 51].

Work from home was an important change implemented in the routine of the population due to the COVID-19 pandemic. To date, very few studies have aimed at understanding the health implications of work from home. Changes in physical behaviors, such as increased sitting and lying down time, and less time spent on PA were expected [52]. However, our study found an association between work from home work as a protective factor for PI. This is in contrast to the findings of a study recently conducted in Brazil, in which office workers were found to have reduced their PA practice in leisure time when comparing the periods before (office workday, September 2019) and during the pandemic (work from home workday, July 2020) [52]. Additionally, a longitudinal study of 112 office workers in the United States, initiated in January 2018, immediately before (i.e., February 2020) and during the detachment of COVID-19 (i.e., at the time of survey data collection), recorded no significant changes in PA during leisure [53]. The authors of the present study, suggest that working at home can create opportunities for individuals to become physically active. It reduces the time spent commuting to work and/or school, in addition to reducing time spent on the workday, providing flexible schedules and additional time to engage in PA. This association is also influenced by the profile of individuals who worked at work from home, with a higher proportion having higher income and more education.

The main limitations of this study are the variables obtained by self-report, which may lead to underestimation of risk behaviors or overestimation of protective behaviors. The study design did not allow for the causality assessment. Furthermore, residual confounding by unmeasured factors cannot be completely excluded. However, several confounders associated with dependent variables were adjusted. Furthermore, the outcome variable was evaluated only in a binary way if individuals engaged in PA during leisure time. Thus, we did not evaluate the frequency, duration, and type of PA modality, limiting the obtainment of information about the individuals’ level of engagement, according to the world recommendations on PA. However, it is important to point out that, to the best of our knowledge, our study is one of the first investigations on PI at different times of the COVID-19 pandemic in Brazil. This outcome variable is among the top five risk factors for increased chronic disease occurrence and mortality worldwide, accounting for up to 10% of the global burden of coronary heart disease, type 2 diabetes, breast and colon cancer, and premature mortality [54]. Therefore, information about the impacts of the COVID-19 pandemic on PI is valuable and contributes to the planning and targeting of population health promotion actions. Therefore, it is important to understand the changes in PI during the period of restricted social movement, so that the negative effects can be reversed, from the creation and implementation of effective and feasible policies to increase PA at all population levels. In addition, we highlight that the data of the present study were derived from a population survey conducted during the pandemic, which is an important source of information on the sanitary situation and health determinants. Additionally, probabilistic sample selection and sample weight provided statistical power to the study, as well as internal and external validity.

## Conclusions

These results suggest that the disruption of daily routine due to the COVID-19 pandemic may have negatively influenced the practice of leisure-time PA, substantially increasing the prevalence of PI in the first months of the pandemic (M1), a condition that remained at M2. We also found that during the COVID-19 pandemic, individuals with lower education, overweight status, and obesity were more likely to be physically inactive, and work from home work favored PA.

## Data Availability

The datasets generated and/or analyzed as part of the current study are not publicly available due to confidentiality agreements with subjects. However, they can be made available solely for the purpose of review and not for the purpose of publication from the corresponding author upon reasonable request.

## Abbreviations

PA: Physical activity
PI: Physical inactivity
M0: Before the pandemic
M1: From March to July 2020)
M2: From October to December 2020
WHO: World Health Organization
SARS-CoV-2: Severe acute respiratory syndrome coronavirus 2
BMI: Body mass index
OR: Odds ratios
CI: Confidence intervals (95%)
MW: Minimum wage
